# Free-ranging squirrels perform stable, above-branch landings by balancing using leg force and nonprehensile foot torque

**DOI:** 10.1242/jeb.249934

**Published:** 2025-04-04

**Authors:** Sebastian D. Lee, Stanley Wang, Duyi Kuang, Eric K. Wang, Justin K. Yim, Nathaniel H. Hunt, Ronald S. Fearing, Hannah S. Stuart, Robert J. Full

**Affiliations:** ^1^ Department of Mechanical Engineering, University of California at Berkeley, Berkeley, CA 94720-1740, USA; ^2^ Department of Mechanical Engineering, Stanford University, Stanford, CA 94305, USA; ^3^ Department of Organismic and Evolutionary Biology, Harvard University, Cambridge, MA 02138, USA; ^4^ Department of Mechanical Engineering, Massachusetts Institute of Technology, Cambridge, MA 02139, USA; ^5^ Department of Mechanical Science and Engineering, University of Illinois Urbana-Champaign, Urbana, IL 61801, USA; ^6^ Department of Biomechanics, University of Nebraska Omaha, Omaha, NE 68182, USA; ^7^ Department of Electrical Engineering and Computer Science, University of California at Berkeley, Berkeley, CA 94720-1740, USA; ^8^ Department of Integrative Biology, University of California at Berkeley, Berkeley, CA 94720-1740, USA

**Keywords:** Locomotion, Arboreal, Jumping, Landing, Balance control, *Sciurus niger*

## Abstract

For gap-crossing agility, arboreal animals require the ability to stabilize dynamic landings on branches. Despite lacking a prehensile grip, squirrels achieve stable landings using a palmar grasp. We investigated the landing dynamics of free-ranging fox squirrels (*Sciurus niger*) to uncover strategies for stable, above-branch landings. Using high-speed video and force-torque measurements in the sagittal plane, we quantified landing kinetics across gap distances. Squirrels rapidly managed >80% of the landing energy with their forelimbs. With larger gaps, peak leg force and foot torque increased. Alignment between forelimbs, velocity and force also increased, likely reducing joint moment. We tested control hypotheses based on an extensible pendulum model used in a physical, hopping robot named Salto. Squirrels stabilized off-target landings by modulating leg force and foot torque. To correct for undershooting, squirrels generated pull-up torques and reduced leg force. For overshooting, squirrels generated braking torques and increased leg force. Embodying control principles in leg and foot design can enable stable landings in sparse environments for animals and robots alike, even those lacking prehensile grasps.

## Teaser

**Highlighted Article:** Squirrels achieve stable, nonprehensile branch landings by controlling leg forces and foot torques, offering insights for agile robotic design.

## INTRODUCTION

Grasping capabilities can affect locomotion, feeding, social interactions and reproductive behaviors of many animals, including all tetrapod clades (Pouydebat et al., 2023; Sustaita et al., 2013). Among locomotor behaviors in tree canopies, gap crossing onto narrow and sparse branches stands out as a common dynamic activity that often requires high-impact contact and stabilization (Graham and Socha, 2020).

To stabilize dynamic motion, primates can use highly effective prehensile grasps, a type of grasp where appendages wrap around an object. They employ opposable digits such as the pollex (thumb) and hallux (big toe) to navigate arboreal environments (Toussaint et al., 2020; Nyakatura, 2019). While primates have been extensively studied, other tree-dwelling mammals such as squirrels also demonstrate remarkable adaptations for arboreal locomotion. Research on tree squirrel biomechanics has primarily focused on quantifying locomotor dynamics along branches of various inclines and sizes (Dunham et al., 2019; Essner, 2002; Wölfer et al., 2021; Orkin and Pontzer, 2011; Youlatos, 1999; Schmidt and Fischer, 2011).

Young and Chadwell (2020) directly compared the locomotor mechanics along a branch in a sciurid rodent and two platyrrhine primates. These species were selected to represent different evolutionary stages of grasping adaptations in primates, from primitive to more evolved. When presented with branches of different widths, squirrel monkeys, with their superior prehensile abilities, exhibited minimal kinematic adjustments in gait, speed, duty factor and peak impact force. In contrast, marmosets demonstrated moderate adjustments, whereas squirrels, which lack a prehensile grasp, required the greatest adjustments. Interestingly, squirrels were also characterized by the lowest values of peak rolling angular momentum over a stride. Assuming limited grasping ability to apply torques to modulate roll, the results suggest that squirrels effectively used dynamic stability as a control strategy for arboreal balance. In the present study, we hypothesize that foot friction plays a significant role in the landing and pitch balancing mechanism of squirrels, indicating a possible nuanced interplay between dynamic stability and the application of torque about a branch.

To better understand the challenges presented by arboreal environments, we can draw insight from robotic grasp taxonomies, which offer precise analytical frameworks for examining the role of foot friction. Grasp types have been defined by an object's shape and size relative to the gripper and its configuration relative to the object, which may vary in the degree of digit contact and wrapping (Cutkosky and Howe, 1990). Feix et al. (2015) define a power palmar grasp with an adducted thumb as a grasp type that relies on opposability afforded by palm and limited digit wrapping. Remarkably, squirrels employ only nonprehensile palmar grasps yet execute highly precise and stable, above-branch landings, all while maintaining readiness for dynamic parkour-like movements.

Studies on squirrel across-branch locomotion have examined jumping (Hunt et al., 2021; Boulinguez-Ambroise et al., 2023) and gliding (Byrnes et al., 2008; Paskins et al., 2007; Scheibe et al., 2007) dynamics. Hunt et al. (2021) studied free-ranging fox squirrels jumping from simulated branches to narrow perches, revealing sophisticated decision-making processes. Squirrels balanced trade-offs between branch-bending compliance and gap distance when choosing launch points and demonstrated the ability to modify impulse generation upon repeated jumps from unexpectedly compliant beams. Impressively, in over 100 trials, squirrels never missed or fell.

**Fig. 1.**
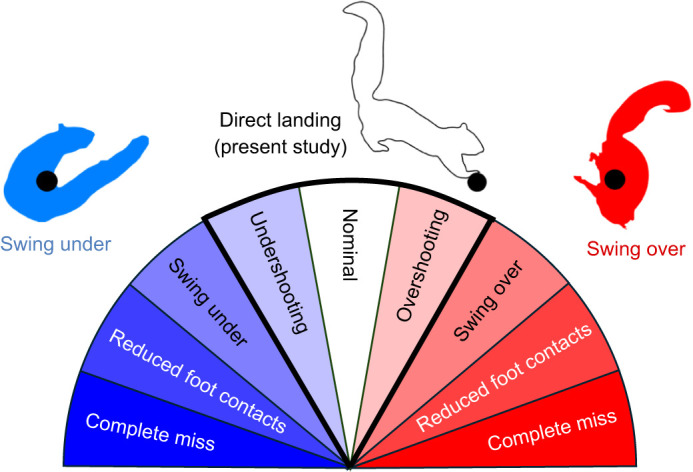
**Types of landing in squirrels.** As defined by Hunt et al. (2021), squirrels can land in a variety of ways. Squirrels may swing under a target branch (dark blue), sometimes being able to make contact with only their front feet (reduced foot contact). Squirrels may also swing over a branch (dark red), sometimes only being able to make contact with their hind feet. Squirrels may also land directly on a branch (middle sectors) and avoid center of gravity (CG) inversion altogether, which is critical for taking another leap if necessary. Within the envelope of direct landings, squirrels may land nominally (zero landing error), undershoot (negative landing error) or overshoot (positive landing error) their target.

When far off-target, squirrels exhibited fail-safe and fault tolerant landings, skillfully swinging their center of gravity (CG) under or over the target branch (Fig. 1). As spectacular as these landings are, unstable landings can impede rapid responses, increasing susceptibility to predation. Despite squirrels using nonprehensile palmar grasps, most branch landings were direct (Fig. 1, three middle sectors) and avoided rotations of the center of gravity under (Fig. 1, darker blue sectors) or over (Fig. 1, darker red sectors) the branch, allowing squirrels to maintain their posture above the branch for subsequent maneuvers. To further understand these abilities, we aimed to quantify the dynamics of direct landings, testing hypotheses of control stabilizing mechanisms that correct for undershooting (Fig. 1, lightest blue sector) and overshooting (lightest red sector) when deviating from nominal (white sector).

**Fig. 2.**
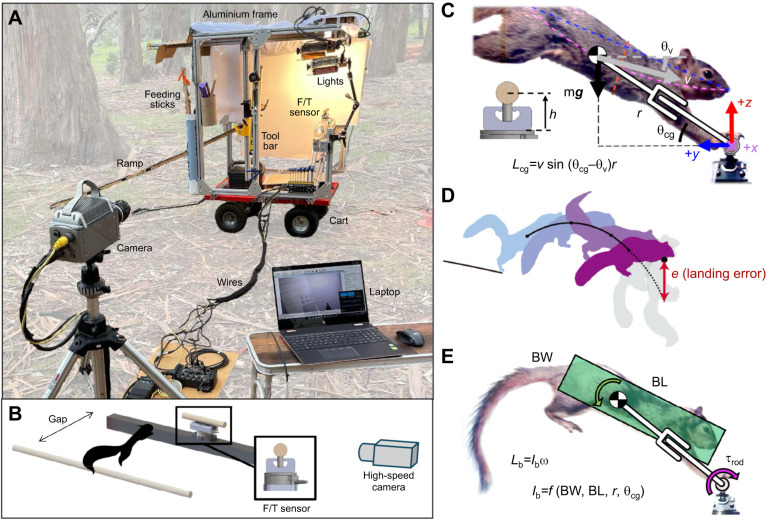
**Field apparatus and key kinematic quantities of *Sciurus niger* landing.** (A) Mobile field apparatus in the eucalyptus forest. High-speed video recordings and force-torque measurements were made during voluntary landings using the cart supporting the aluminum frame and F/T sensor, a ramp, lights, high-speed camera and a laptop for data acquisition. (B) Diagram showing the take-off branch, the gap distance jumped, the force/torque (F/T) sensor (with lateral view in box) and high-speed camera. The take-off rod was attached to a linear rail, which allowed for variable gap distances of 50, 75 and 100 cm. (C) Center of gravity (CG) is graphically calculated by fitting a parabola between three points as in Hunt et al. (2021): nose, tailbase and the midpoint of the ventral-dorsal line (red dashed), which approximately bisects the tailbase–nose line (blue dashed). CG position and velocity were extracted to calculate CG angular momentum (*L*_cg_) and landing error (*e*). The distance, *h*, between F/T sensor interface and the branch axis was *h*=40 mm. (D) Landing error (*e*) was calculated as the projected vertical offset of the extrapolated aerial trajectory (see Hunt et al., 2021). (E) Body angular momentum (*L*_b_) is a function of body moment of inertia (*I*_b_) and body pitch rate (ω). The body is modeled as a cylinder of body length (BL), diameter (body width, BW) and at the CG position. All variables are defined in Table 1.

To quantify nonprehensile, high-impact, dynamic landing in free-ranging squirrels, we conducted experiments in a eucalyptus forest using artificial branches. We measured both touchdown state and forces in the sagittal plane at various gap distances. We used high-speed video to measure the landing kinematics and we designed a force-torque sensor apparatus that could be transported to the field to enable the first landing kinetic measurements on a horizontal branch (Fig. 2A–C). This approach allowed us to test control hypotheses for direct landings postulated by Yim et al. (2025) for the physical model/robot Salto (Haldane et al., 2016; Yim and Fearing, 2018; Yim et al., 2020). We modeled the CG to front foot contact as a compressible virtual leg comparable to a simple extensible pendulum system (Fig. 2E), which provides a conceptual framework for interpreting the results.

**Fig. 3.**
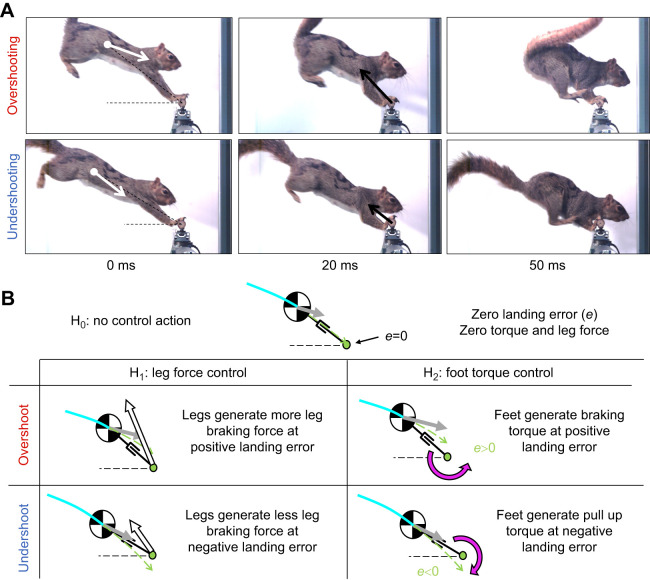
**Balanced landing control hypotheses for undershooting and overshooting trajectories.** (A) Snapshots of overshooting and undershooting at three different time intervals: touchdown, 20 ms and 50 ms. At touchdown, the white arrows begin at the CG and represent the velocity vector relative to the branch (see Movie 1). (B) Force-torque control hypotheses derived from Yim et al. (2025) exemplify the interplay of leg force and foot torque control for balanced, above-branch landings. When overshooting, squirrels apply greater braking forces and torque. When undershooting, squirrels apply lower braking forces and pull-up torques. The green dot represents the branch, and the dashed line, the branch level. The light blue line shows the trajectory prior to touchdown and the green dashed arrow, the projected trajectory. The gray arrow shows the touchdown velocity vector, the white arrow, the branch reaction force, and the magenta arrow, the branch reaction torque direction.

We hypothesized that a nominal, direct landing (Fig. 1, white sector) could be attained by squirrels using only their angular momentum to swing to a stable, balanced position. Far more likely, however, we postulated that squirrels would undershoot or overshoot the target branch (Fig. 3A, Movie 1). Stabilizing off-target landings by controlling rotation about the branch could be accomplished by modulating leg force and foot torque (Fig. 3B). Specifically, control of front leg force could correct for overshooting by generating more leg-braking force, whereas undershooting could be stabilized by generating less leg-braking force. When overshooting, squirrels can also generate braking torques in the direction opposite to their forward motion, countering large landing errors and excessive rotation. Conversely, for undershooting, they can produce a pull-up torque in the direction of their forward motion, correcting for negative landing errors.

In addition to quantifying and characterizing squirrel branch landings, our study also aims to stimulate further research on the role of embodied control in foot and leg design for dynamic, high-impact interactions. Understanding the biomechanics of the elaborate morphology of squirrel foot and toe design can lead to novel use of metamaterials (e.g. Jin et al., 2023). Interdisciplinary collaboration with engineers could provide biological inspiration for the development of the next generation of legs and feet for agile legged robots, as well as nonprehensile robotic manipulators. These findings show that examining dynamic, high-impact landings and stabilization with only nonprehensile, palmar grasps can further advance the field of grasping and manipulation for both animals and engineered systems.

## MATERIALS AND METHODS

To measure squirrel landing dynamics, we designed experiments to capture the kinematics, forces, and torques using an instrumented branch or rod. The experiments involved three key components: (1) the acquisition and training of free-ranging research animals; (2) the utilization of a mobile instrumented apparatus; and (3) a systematic data collection and processing procedure for subsequent analysis. The ethical treatment of animals and adherence to protocols were ensured through the approval of the University of California, Berkeley's Animal Care and Use Committee (ACUC) Protocol #AUP-2018-06-11201-1 and the California Department of Fish and Wildlife Nongame Wildlife Program.

### Animal preparation

*Sciurus niger* Linnaeus 1758 are free-ranging arboreal squirrels known for their adept navigation in tree canopies and have adapted to urban environments. Their agility and accessibility make *S. niger* an ideal candidate for studying and characterizing arboreal locomotion (Hunt et al., 2021). Here, we focus on the measurement of landing forces, rod torque and high-speed video involving four female fox squirrels (729±63 g; mean±s.d.). To uniquely identify each individual, a non-toxic fur dye (Nyanzol D) was prepared and dispersed on the squirrels' fur.

Introducing free-ranging individuals to a novel apparatus required training squirrels with a shaping paradigm using peanuts as positive reinforcement for approximately 3–5 weeks. Individuals were introduced to the setup at different times over a span of 4 months based on their voluntary willingness to cooperate. After acclimation, individuals were trained to follow a feeding stick and execute jumps from a non-instrumented take-off birch perch to an instrumented target birch perch [both 1.91 cm (0.75″) diameter]. To ensure consistent behavior meeting our operational definition of direct landing, each individual underwent a training period consisting of at least five successful landings on the instrumented perch, before data collection. A successful landing was defined as a direct landing in which foot contact is restricted to the rod only without swinging under or over the landing target (Fig. 1, center sectors outlined in bold). Training was administered to each individual at each gap distance and was then followed by five more landings for which data were collected.

### Field force, torque, and kinematics apparatus

We designed and implemented an experimental setup to simultaneously capture three-dimensional force/torque data and 2D high-speed video of squirrels landing on artificial and instrumented branches. The force measurement device (Fig. 2A,B) utilized a 6-axis force/torque (F/T) transducer (ATI Mini45 with SI-145-5 calibration) and data acquisition system (NI USB-6210 DAQ). The F/T sensor was connected to the DAQ and a power supply for 3D force data streaming. The DAQ was connected to a computer (Windows 11 Laptop) for data recording through NI LabVIEW. The hardware selection allowed for measurements with force resolution of 62.5 mN in all three axes, a torque resolution of 1.3 N mm in the *X*-axis and a sampling rate of 3000 Hz.

The mechanical structure of the apparatus was designed for high stiffness but also to be lightweight and compact for ease of transport into the field (Fig. 2A). To minimize vibrations in the F/T signal, an aluminium truss was attached to the base-side of the F/T sensor. An artificial branch was attached to the topside of the sensor, and its offset from the sensor interface was minimized to maximize the cantilever's natural frequency, which aided in minimizing signal loss when filtering the signal. Outdoors, the ground can be uneven, so two measurement levels were attached to the setup to ensure the sensor was as horizontal as possible. The take-off branch was attached to a rail, which enabled quick adjustment of gap distance, from 50 cm to 100 cm, where 100 cm is approximately four squirrel body lengths.

As shown in Fig. 2A, the instrumented setup was transported to a nearby eucalyptus grove. A ramp guided squirrels to the take-off branch substrate. A high-speed camera (Vision Research, Phantom v. 10.0) sat level and approximately 2.5 m away from the subject. Flood lights (Lowel Tota-Light Tungsten) provided a non-flickering light source. The high-speed camera was set to record at 500 frames s^−1^ with a resolution of 1920×1080 pixels. Force and video data were electronically synchronized through the Vision Research GUI.

Instruments of the apparatus (i.e. camera, flood lights, laptop, DAQ) were first connected to an outdoor power outlet. The F/T sensor and camera were placed and then leveled such that the landing substrate and squirrel's body were visible in the camera frame. Given the brevity of the landing event, the instruments were armed to end-trigger recordings. When available, free-ranging individuals were guided towards the setup with feeding sticks. A researcher prompted an individual to climb up the ramp and to cross the gap by leaping between rods or perches. For a given trial, another researcher end-triggered force and video data as soon as the squirrel came into contact with the F/T sensor. Data were then written to the laptop. After five trials per individual, data collection was terminated for the given experimental condition.

### Feature extraction

We digitized the high-speed video sequences of each landing by manually marking key points and drawing lines between them as shown in Fig. 2C. Their motion was tracked for at least 5 frames pre- and post- front feet touchdown (±10 ms from *t*_0_) to ensure a reliable estimate for landing velocity. These key points were chosen from a previous study (Hunt et al., 2021) and were used to calculate CG in the same manner. Key tracked features included the nose, tail base, dorsal midpoint, ventral midpoint and branch center. We drew a transverse line between the dorsal and ventral midpoints. Then, we fitted a parabola between the midpoint of the transverse line, the nose and tail base. The trajectories of these points were computed in the camera view. The CG position and velocity were parametrized by: *r* (leg length), *v* (CG speed), θ_cg_ (leg angle) and θ_v_ (velocity angle) as defined in Table 1. From these parameters, we also computed radial and tangential speeds:
(1)

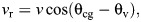

(2)

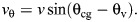
From *r*, *v*, θ_cg_ and θ_v_, we also calculated landing error and total angular momentum (Fig. 2C–E). Landing error, *e*, was computed as:
(3)

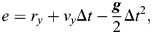

where Δ*t*=*r_x_*/*v_x_*, the time required to travel a distance *r_x_* at speed *v_x_*. *r_x_*, *r_y_*, *v_x_* and *v_y_* are the horizontal and vertical components of leg length and velocity, respectively, and ***g*** is gravitational acceleration.

**Table 1.** Measured and calculated times, force/torque and kinematic variables used for characterizing each landing sequence

Variable	Name	Unit	Description
Timing			
*t*_1_	1st peak time	ms	First peak force
*t*_hf_	Hind feet touchdown	ms	Second contact
*T*	Settling time	ms	End of landing
Force, torque, impulse			
*F*_h_	Horizontal force magnitude	N	*y*-axis force
*F*_v_	Vertical force magnitude	N	*z*-axis force
τ_x_	*x*-axis torque magnitude	N mm	Measured from F/T sensor
*F*_net_	Net force magnitude	N	Camera-view force 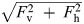
θ_F_	Force angle w.r.t. horizontal	deg	atan2(*F*_v_, *F*_h_)
τ_rod_	Rod-axis torque magnitude	N mm	τ_rod_=τ_*x*_−*F*_h_*h*, where *h* is defined in Fig. 2C
*J*_0_	Impulse from front legs	N s	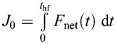
*J*_T_	Total landing impulse	N s	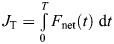
Touchdown kinematics			
*r*	Virtual leg length from rod to CG	mm	Distance *r* in Fig. 2C
*v*	CG speed	m s^−1^	Speed *v* in Fig. 2C
			
θ_cg_	CG touchdown angle w.r.t. horizontal	deg	Angle θ_cg_ in Fig. 2C
θ_v_	Velocity angle w.r.t. horizontal	deg	Angle θ_v_ in Fig. 2C
BL/BW	Body length and width	mm	Dimensions of squirrel body as a cylinder
*I*_b_	Body moment of inertia	m²	*I*_b_=*f* (BW, BL, *r*, θ_cg_), see Materials and Methods
γ	Body pitch	deg	Angle w.r.t. horizontal of blue dashed line in Fig. 2C
ω	Body pitch rate	deg s^−1^	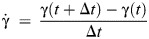
*e*	Landing error	mm	Distance *e* in Fig. 2D
*L*_cg_	CG angular momentum	m² s^−1^	*L*_cg_=*v* sin(θ_cg_ -θ_v_)*r* in Fig. 2C
*L*_b_	Body angular momentum	m² s^−1^	*L*_b_=*I*_b_ω
*L*_total_	Total angular momentum	m² s^−1^	*L*_total_=*L*_cg_+*L*_b_

CG and body angular momentums were computed as:
(4)

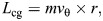

(5)

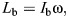
where *I*_b_ is the instantaneous body moment of inertia and ω is the body pitch rate calculated as:
(6)

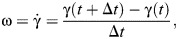
where γ is the body pitch. We approximated the squirrel as a cylinder with diameter BW and length BL. Then, the moment of inertia can be approximated as:
(7)

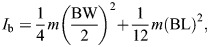
where *m* is the squirrel's mass (measured with force sensor when at rest), BW is the squirrel's body width, and BL is the body length. The sum of angular momentums yields the total angular momentum:
(8)

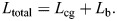


From the synchronized dataset, we filtered the raw force data from each trial to remove high-frequency noise and mechanical vibrations from the setup. We used an 8th order forward–backward low-pass Butterworth filter with a 40 Hz cutoff frequency. Filter design and minimal attenuation were facilitated by the fact that both the F/T sensor attachment and base have sufficiently high natural frequencies in the vertical and horizontal axes (above 300 Hz). When a squirrel comes into contact with the setup, however, the natural frequency of the squirrel–branch system decreases significantly. Through signal mode decomposition, we found a cutoff frequency of 40 Hz was a good balance between removing system vibrations and preserving the signal of interest. Features were extracted from net force and rod torque data as defined in Table 1. Rod torque was computed with the following equation:
(9)

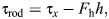
where τ*_x_* is the raw torque measurement about the *X*-axis, *F*_h_ is the raw horizontal force measurement, and *h* is the distance between the branch center and the F/T sensor's interface. Forelimb and total linear impulse were computed as follows:
(10)

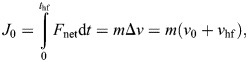

(11)

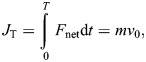
where *F*_net_ is the net force, *t*_hf_ is the hindfoot touchdown time, *v*_hf_ is the unknown speed at *t*_hf_ and *T* is the time to landing completion. Impulse can be approximated as the mass times change in speed for the given time intervals. Then with these two impulses, we defined forelimb impulse contribution α, which yields a relationship between α, *v*_0_ and *v*_hf_:
(12)

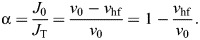


We also define energy states at *t*=*t*_0_ and *t*=*t*_hf_:
(13rmA13rmB)

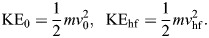


We define forelimb energy contribution β using these two energy states, which yields a relationship between β, *v*_0_ and *v*_hf_:
(14)

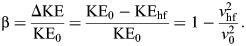
Combining the equations above yields the relationship between α and β,
(15)

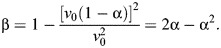


### Statistical analyses

Over eight experimental sessions, squirrels performed 60 successful landings. We focused our analysis on force and high-speed video data of *n*=60 successful landings, five trials for four individuals at three gap conditions. Some trials were excluded for one of two reasons: (1) The camera view did not capture the squirrel's tail base at touchdown, so CG kinematics at touchdown could not be extracted (*n*=11), or (2) the squirrel severely undershot or overshot the target branch, resulting in an unstable landing, appendages coming into contact with other parts of the setup, and therefore invalid force-torque measurements (*n*=2). Then, for extraction of the landing state from high-speed video data, we analyzed *n*=49 landings. Means and standard deviations are reported for metrics across all trials for each gap condition. *P*-values and *F*-statistics are reported from one-way repeated-measures ANOVA comparisons across gap distances. *P*-values and t-statistics are also reported for the linear mixed-effects models controlling for gap and individual to show predictive power of landing error and angular momentum. Data were analyzed using MATLAB statistical software tools.

## RESULTS

### Landing dynamics as a function of time

#### Kinematics of landing

**Fig. 4.**
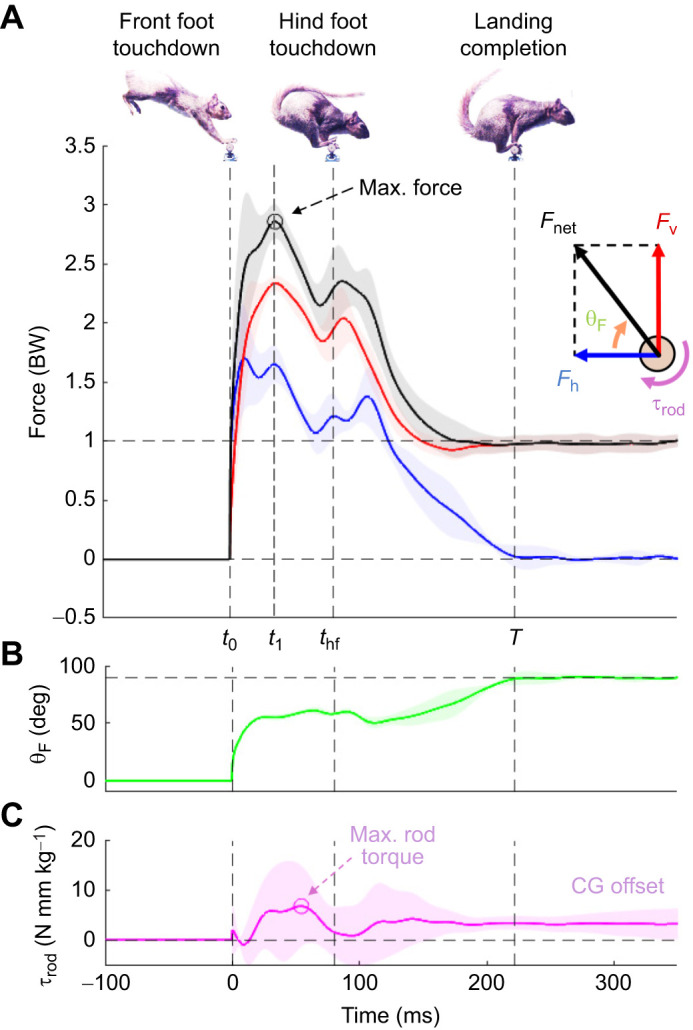
**Branch landing reaction force, maximum force angle and rod torque as a function of time for a given gap and individual (*n*=5).** (A) Mean net force and force components plotted over time. Shaded bands show one standard deviation. Squirrels showed maximum force peaks during front feet touchdown and a secondary smaller peak after hind feet touchdown. Vertical dashed lines indicate critical landing times (*t*_1_, time to peak force; t_hf_, hind feet touchdown; *T*, landing completion time). Front feet (*t*_0_) or hind feet (*t*_hf_) touchdown time is defined as the frame in which the feet first come into contact with the branch. Settling time (*T*) is defined as the time at which *F*_h_ reaches 0 N. (B) Net force angle stabilizes at approximately 50 deg and steadily increases to a vertical 90 deg after *t*_hf_ until *t*=*T*. (C) Maximum rod torque can be positive and negative throughout the time series, as seen in the magenta shaded area. When *t*>*T*, τ_rod_ settles to a non-zero value, which corresponds to static torque while perched.

Throughout the experiments, squirrels crossed gaps following projectile motion trajectories. Forces normal to the camera view (*X*-axis of the load cell) were negligible compared with forces in the squirrel's sagittal plane (*Y–Z* plane of load cell). Squirrels exhibited a diverse range of landing touchdown states that varied in CG position and velocity with respect to the branch. In all landings, squirrels always touched down with their front feet first, quickly followed by their hind feet (Fig. 4A). Upon landing, squirrels actively controlled their body pose by rotating their joints so that their hind legs could contact the branch. Contact with their hind feet was required to reach the stable, above-branch, perched state. In a typical sequence for a given gap and individual, squirrels prepared for touchdown by extending their forelegs and front feet. The extended feet first engaged with the branch to create a reliable anchor. Then, the front limbs compress as kinetic energy decreases. A force peak always followed front feet touchdown. The first peak force induced by the front limbs was always greater than the second one, which was induced after hind feet touchdown. At the end of the landing at *t*=*T*, the hind feet provided friction for static balance while the front feet either stayed in contact or detached.

#### Branch reaction force timing during landing

Landings for a given individual were consistent. A characteristic set of leg reaction force data (*n*=5 trials) for one individual at the 75 cm gap distance is shown in Fig. 4A with its net force angle in Fig. 4B. Fig. 4C illustrates rod torque over time and highlights the maximum rod torque, which on average was positive (in the pull-up direction). Here, the shaded regions illustrate variability, which is defined as the standard deviation across five trials. We define *t*_0_ as *t*=0 ms, the time at which the front feet first come into contact with the branch. We visually examined high-speed video to extract the frame at which contact occurred. The first force peak occurred at *t*_1_, the time at which *F_net_* reached its global maximum. The hindfoot touchdown time, *t*_hf_*,* is defined as the time at which the hind feet first make contact, which was visually noted on the high-speed video. Finally, we estimated the end of the landing, *T*, as the time at which the horizontal force, *F*_h_, reached and settled to 0 N. Out-of-plane peak forces were ignored, as they were on average 1.34±0.69 N (or 0.19±0.09 BW). This was on average 8.7% of observed *F*_net_. Snapshots corresponding to *t*_0_, *t*_hf_ and *T* are displayed sequentially in Fig. 4A. Table 2 reports time variables mean and standard deviation for each event at each gap distance tested. Depending on gap distance, squirrels completed landings within *T*=200–350 ms. Squirrels reached peak forces within 20–65 ms. Hind feet touchdown occurred within 60–140 ms or within 29–43% of *T*.

**Table 2.** Landing variable measurements as a function of gap distanced

	Gap distance				
	50 cm	75 cm	100 cm	*F*	*P-*value
Timing					
*t*_1_ (ms)	45±20	28±7.7	22±9	30.8	8.04e^–7^ ***
*t*_hf_ (ms)	124±15	95±12	68±10	353.9	2.30e^–26^ ***
*T* (ms)	313±61	262±27	232±59	30.6	7.97e^–7^ ***
Force and torque					
*F*_net_/*m**g*** (BW)	2.09±0.19	3.25±0.30	4.31±0.49	488	2.51e^–29^ ***
θ_F_ (deg)	51.7±3.4	47.5±4.4	46.1±5.2	38.3	7.47e^–8^ ***
τ_rod_/*m* (N mm kg^−1^)	54±58	95±97	136±169	5.0	0.029 *
|τ_rod_|/*m* (N mm kg^−1^)	72±31	114±73	191±97	31.3	6.86e^–7^ ***
Impulse					
*J*_0_ (N s)	1.09±0.29	1.64±0.34	1.77±0.32	81.4	1.65e^–12^ ***
*J*_T_ (N s)	1.67±0.30	2.33±0.35	2.77±0.26	324.7	5.66e^–25^ ***
α (%)	65.4±9.7	70.3±8.3	64.1±11.3	0.3	0.58
β (%)	87.1±7.6	90.5±5.5	85.9±9.0	0.5	0.48
Touchdown kinematics					
*r* (mm)	205±9	208±10	208±7	5.7	0.021 *
θ_cg_ (deg)	34.9±4.0	34.4±4.8	37.7±5.6	2.7	0.10
θ_v_ (deg)	23.1±4.8	28.4±4.9	34.1±4.7	145.4	5.46e^–16^ ***
*v* (m s^−1^)	1.71±0.18	2.29±0.23	2.79±0.18	251.7	1.68e^–20^ ***
*v*_r_ (m s^−1^)	1.67±0.17	2.28±0.23	2.78±0.18	277.2	2.44e^–21^ ***
*v*_θ_ (m s^−1^)	0.35±0.09	0.24±0.10	0.17±0.15	32.6	7.45e^–7^ ***
BL (mm)	279.2±16.3	270.7±13.1	270.0±7.8	4.7	0.035 *
γ (deg)	24.3±3.3	26.7±3.7	25.4±3.7	0.4	0.51
ω (deg s^−1^)	−122.4±63.2	−59.6±87.1	−25.0±84.2	14.0	5.06e^–4^ ***
Landing error and angular momentum					
*e* (mm)	−11.7±18.2	−11.9±15.0	−9.7±16.5	0.6	0.43
*L*_cg_ (m^2^ s^−1^)	0.073±0.020	0.051±0.023	0.036±0.031	30.6	1.38e^–6^ ***
*L*_b_ (m^2^ s^−1^)	−0.015±0.009	−0.007±0.010	−0.003±0.009	15.0	3.37e^–4^ ***
*L*_total_ (m^2^ s^−1^)	0.058±0.016	0.044±0.023	0.033±0.034	10.5	2.20e^–3^ **

BL, body length; α, forelimb impulse contribution; β, forelimb energy contribution; *v*_r_, radial speed; *v*_θ_, tangential speed. For all other variables, please refer to Table 1. *F* statistics and *P*-values are reported from repeated-measures ANOVA comparisons between experimental conditions. *P<0.05; **P<0.01; ***P<0.001.

#### Impulse reflecting energy management by forelimbs

Forelimb impulse is calculated as the area under the *F*_net_ curve from *t*_0_ to *t*_hf_ (see Table 1). *J*_T_ is the total impulse induced by the landing event. Impulse can also be calculated as a change in momentum (*J*=*m*Δ*v*). We define α as the ratio between forelimb impulse and total impulse, α=*J*_o_/*J*_T_ (Eqn. 12). We found that α=67±10%, which means on average, 67% of speed was decelerated by the forelimbs alone.

We can also compute β, the ratio between kinetic energy managed by the forelimbs over the total touchdown kinetic energy. Here, we express β in terms of α, as β=2α–α^2^ (Eqn 15). Using this equation, we calculate that on average, squirrel forelimbs managed 88% of landing kinetic energy, regardless of gap distance. Forelimb energy absorption occurred within 60–130 ms, which on average was 36% of *T*. Therefore, forelimbs were responsible for managing most of the landing kinetic energy in a fraction of the landing period.

### Landing dynamics as a function of gap distance

#### Gap distance effect on touchdown kinematics

Squirrel touchdown kinematics are defined by the CG's position and velocity at t_0_. As seen in Table 2, virtual leg length (*r*) and leg angle (θ_cg_) did not vary significantly across gap distances (*P*>0.01). However, speed (v) and velocity angle (θ_v_) both increased with gap distance. As gap distance increased, squirrels took higher (one-way repeated measures ANOVA, *F*_1,47_=145.4, *P*<0.001) and faster ballistic trajectories (*F*_1,47_=251.7, *P*<0.001). As gap distance increased, squirrel landing velocity angle, θ_v_, also approached the 45 deg launch angle that tends to maximize horizontal travel for a particular speed.

Touchdown velocity can be broken down into radial and tangential components. Like speed, radial speed (*v*_r_) increased with gap distance (*F*_1,47_=277.2, *P*<0.001). Tangential speed (*v*_θ_) on the other hand, decreased with gap distance (*F*_1,47_=32.6, *P*<0.001). Squirrel touchdown kinematics are also defined by the body's posture and rotational speed at *t*_0_. Body length (BL) and body pitch (γ) were not significantly different across gap distances (*P*>0.01). However, body pitch rate (ω) decreased in magnitude as gap distance increased (*F*_1,47_=14.0, *P*<0.001).

For all key time points, average time decreased as gap distance increased, pointing towards a consistent force behavior over time. Hindfoot touchdown time (*t*_hf_) decreased from 124 ms at 50 cm to 68 ms at 100 cm, indicating hind feet touched down faster at longer gap distances (*F*_1,58_=353.9, *P*<0.001; see Table 2). The landings were completed more rapidly the longer the gap distance (*F*_1,58_=30.6, *P*<0.001). The landing sequences themselves were approximately 80 ms shorter at the 100 cm gap compared with the 50 cm gap. Time to peak reaction force also occurred sooner, decreasing from 45 ms to 22 ms (*F*_1,58_=30.8, *P*<0.001).

#### Gap distance effects on force-torque, force angle, and impulse

**Fig. 5.**
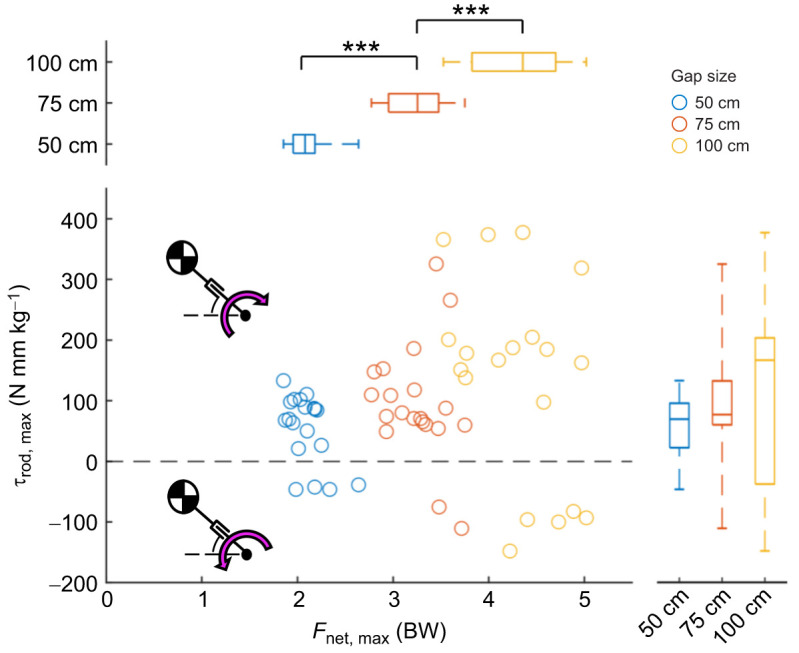
**Grasping wrench space represented by peak foot torque (τ_rod_) and peak leg force (*F*_net_) at each gap distance.** Wrench space is the set of force and torque combinations that permit dynamic landing. Individual points and box plots illustrate data spread with median. Bars represent quartiles. Peak *F*_net_ magnitude and variation tend to increase with gap distance (***P<0.001). Comparisons are made across gaps (***P<0.001). Mean peak τ_rod_ is statistically different across gaps (*P*<0.05, see Table 2). This difference is more significant when comparing magnitudes of rod torque (***P<0.001, see Table 2). Standard deviation also increases with gap distance, where a Levene's test on torque yielded *P*<0.05. Pink arrow, branch reaction torque direction.

Upon touchdown across all trials, squirrels exerted a peak wrench (peak force and torque) on the branch (Table 2). As expected, peak *F*_net_ increased with gap distance more than doubling from 2.09 BW at 50 cm to 4.31 BW at the 100 cm distance (Fig. 5, *F*_1,58_=488, *P*<0.001). Maximum force angle (θ_F_) decreased with increasing gap distance (*F*_1,58_=38.3, *P*<0.001) from 51.7 deg at 50 cm to 46.1 deg at 100 cm (see Table 2), becoming more horizontal at longer gap distances. A lower maximum force angle implies that at longer gap distances, squirrels are relying more on horizontal force rather than vertical force to decelerate their landings.

Peak τ_rod_ magnitude increased with gap distance (*F*_1,58_=31.3, *P*<0.001) more than doubling from 72 N mm kg^−1^ at 50 cm to 191 N mm kg^−1^ at 100 cm (Fig. 5). The range of peak τ_rod_ magnitude values also increased with gap distance, tripling from 31 N mm kg^−1^ at 50 cm to 97 N mm kg^−1^ at 100 cm (see Table 2; Fig. 5). A Levene's test revealed significant heterogeneity of variances across gap distances (*P*<0.05). We postulate that the increasing variation in peak force and torque can be explained by the variation in touchdown state.

Forelimb impulse *J*_0_ and total impulse *J*_T_ increased with gap distance (*F*_1,58_=81.4, *P*<0.001 and *F*_1,58_=324.7, *P*<0.001, respectively; see Table 2). However, forelimb impulse and energy contribution, α and β*,* did not vary significantly across gap conditions (*F*_1,58_=0.3, *P*=0.58, and *F*_1,58_=0.5, *P*=0.48, respectively) indicating that the variation in forelimb contribution is not explained by gap distance. Forelimb impulse contribution (*α*) was 66.7±10.0% and forelimb energy management (β) was 87.9±7.6%.

#### Gap distance effect on landing error and angular momentum

The touchdown kinematics can be simplified to two variables: landing error and total angular momentum. Landing error is defined as the vertical distance between the projected ballistic trajectory of the squirrels' CG and the branch center. On average, landing error across all trials was −11.3±16.4 mm and did not increase or decrease with gap distance (*P*>0.05; Table 2). Normalizing using squirrel body length did not result in statistical differences in landing error.

Total angular momentum is defined as the sum of CG angular momentum, *L*_cg_, and body angular momentum, *L*_b_. Angular momentum of the CG about the branch was significantly different across gap distances (*F*_1,49_=30.6, *P*<0.001), decreasing from 0.073 m^2^ s^−1^ at 50 cm to 0.036 m^2^ s^−1^ at 100 cm. Similarly, body angular momentum decreased significantly in magnitude with increasing gap distance (*F*_1,49_=15.0, *P*<0.001) from −0.015 m^2^ s^−1^ at 50 cm to −0.003 m^2^ s^−1^ at 100 cm. Finally, total angular momentum also tended to decrease with increasing gap distance (*F*_1,49_=10.5, *P*<0.01) decreasing from 0.058 m^2^ s^−1^ at 50 cm to 0.033 m^2^ s^−1^ at 100 cm. Angular momentum was the lowest at the 100 cm gap distance.

## DISCUSSION

### Reaction force patterns and magnitudes during landing

Boulinguez-Ambroise and colleagues (2023) highlight in their study on tree squirrel jumping that, although jumping performance is often discussed as a pivotal aspect of early primate evolution, its quantification in arboreal mammals is lacking compared with other locomotor behaviors, such as quadrupedal walking and running on branches. The prevalent focus on locomotion along branches (Young, 2023; Young and Chadwell, 2020; Wölfer et al., 2021; Dunham et al., 2019; Hesse et al., 2015; Lammers and Gauntner, 2008; Orkin and Pontzer, 2011; Schmidt, 2011) largely stems from the research in the initial stages of primate evolution, particularly concerning the ability to grasp thin terminal branches. Our study extends beyond assessing movement along branches or saltatorial ability by examining the challenge of executing a stable landing on a narrow branch without the advantage of a prehensile grasp.

Since Bonser's (1999) review, surprisingly few studies involve landing kinetics that consider grasping or balancing. Among the studies that have explored landing dynamics, various animals such as birds, lemurs, cats and toads have been investigated. In our fox squirrels (∼750 g), balanced branch landing most often produced a bimodal branch reaction force pattern (Fig. 4A). Within just 20–65 ms after touchdown, squirrels reached peak reaction forces resulting from front foot touchdown. Hind foot touchdown occurred within 60–140 ms, corresponding to a second, smaller reaction force peak. Peak *F*_net_ increased with gap distance, more than doubling from 2.1 BW (multiples of body weight) at 50 cm to 4.3 BW at the 100 cm distance (Fig. 5; Table 2). Peak branch torque τ_rod_ produced by the palmar grasp of the front feet occurred between their front foot touchdown and hind foot touchdown. Average peak τ_rod_ also increased with gap distance, more than doubling (72 N mm kg^−1^ at 50 cm to 191 N mm kg^−1^ at 100 cm; Fig. 5).

Birds, such as parrotlets, demonstrate an intricate use of their feet during landing on branches, employing opposable digits for effective grasping. While birds primarily utilize their wings to generate supportive aerodynamic forces, Roderick et al. (2019) observed that parrotlets (∼30 g) still exert perch reaction forces ranging from 4 to 5 BW within 5–10 ms. Unlike squirrels, which use both front and hind legs for landing, parrotlets use only their legs to apply ground reaction forces, resulting in a unimodal force pattern. Their feet follow a consistent sequence of movements when landing, including spreading, opening, pre-shaping, wrapping around the branch, and curling their claws. Their study revealed that after touchdown, the dynamics of the foot, toes and claws are crucial for a successful perch, and that these anchoring mechanisms are surface specific. These actions, particularly the toe squeeze, enhance stability upon landing, providing an advantage that squirrels lack.

Landing kinetics have been measured in leaping lemurs (∼1–5 kg) attempting to grasp a compliant vertical pole acting as a force sensor (Demes, et al., 1995, 1999). Peak landing reaction forces ranged from 5 to 11 BW, but no time course was reported. These studies show that some lemurs display a more diverse landing pattern and behavior when leaping down to a flat and horizontal force plate. They show a bimodal reaction force pattern representing the front and hind limbs (Demes et al., 2005). However, the first peak reaction force depended on whether the fore- or hindlimb struck the platform first, which differed among the two species in question. Peak vertical reaction forces ranged from 1.7 to 1.9 BW for the forelimb lander (1.9 kg) and 2.0 to 3.1 BW for the hindlimb lander (3.1 kg), both increasing with jump distance. Hindlimb landing species reached their first force peak at 100 ms and completed landing in 400 ms.

Landing kinetics using extending forelimbs, as shown by our squirrels, have also been measured in cats landing on flat surfaces instrumented with a force plate. Cats (4–5 kg) jumping down from a platform extend their forelimbs for landing impact (Zhang et al., 2014b), which is similar to our observations in squirrels. Cats generate a bimodal reaction force pattern that results from the first peak force from front leg landing followed by the rotation of the body, allowing the hind limbs to produce a second peak force (Wu et al., 2019). The bimodal pattern mirrors what we observed in squirrels, though the relative magnitudes differ. As jump height is increased further, peak reaction forces from the front limb increased from approximately 3 to 8 BW, whereas hind limb forces could increase far more, ranging from 2.5 BW to as high as 20 BW (Zhang et al., 2014a). A faster increase in hindlimb peak force implies that as platform height increases, cats use their hindlimbs more for energy management. Correspondingly, their time to peak force shifts from 10 ms to 40–50 ms. Although not tested in our study, it is likely that at comparably demanding gap distances, squirrels might increasingly rely on their hindlimbs.

Similarly, as hopping toads (250 g) prepare for landing, they also fully extend their front legs or hands (Azizi et al., 2014; Reilly et al., 2015; Cox and Gillis, 2017). Like cats and squirrels, toad landing consists of two phases defined by two peaks of vertical reaction force coinciding with the impact of two body parts. In the hand landing phase, the extended arms hit, flex and rotate as they absorb the landing energy. The vertical force peaks tend to be near 1.75 BW. The body/feet landing phase begins when the folded hindquarters (i.e. pelvis, abdomen and feet) hit the ground simultaneously. The peak vertical force for the latter landing phase was approximately equal to body weight, suggesting that, as in squirrels, the toads' forelimbs manage a significant amount of landing energy.

### Force, velocity and leg alignment

Alexander (1991) emphasized the importance of aligning ground reaction force (GRF) vectors with the center of mass and joint centers for energy conservation in legged locomotion. Chen et al. (2006) and Full et al. (1991) demonstrated that animals maintaining a consistent average speed, regardless of their number of legs or posture, tend to align the force vectors along their legs. Vector alignment minimizes joint moments and reduces the work required by the limbs. The significance of alignment can become even more critical during landings at higher speeds, where the primary concern shifts from energy conservation to injury prevention – from efficiency to safety.

**Fig. 6.**
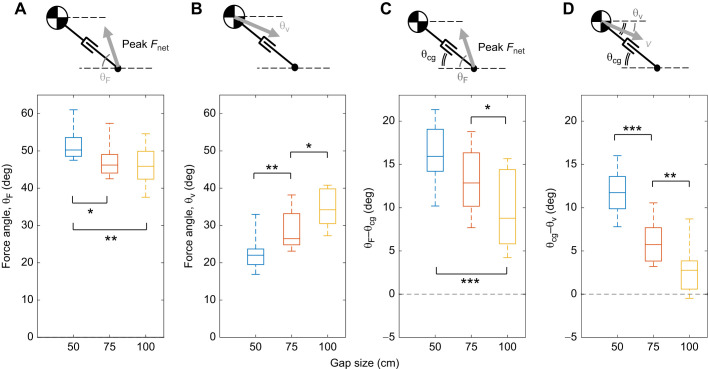
**Force and velocity angles and angles relative to the leg at each gap distance.** (A) Maximum force angle decreases as gap distance increases (*P<0.05, **P<0.01). (B) Velocity angle increases as gap distance increases. (C) Difference between maximum force and leg angle decreases as the gap distance increased (***P<0.001). (D) Difference between velocity and leg angle also decreases. As gap distance increases, maximum force and velocity align more to the forelimb such that Δθ→0.

For landing squirrels, we used impulse estimates to conclude that the forelimbs and torso manage 80–95% of kinetic energy within 60–140 ms (Table 2). We also measured the angle differences between maximum reaction force angle, velocity angle and leg angle (Fig. 6). We found that the difference between the maximum reaction force angle and leg angle decreased with gap distance (Fig. 6C). The difference between leg angle and velocity angle also decreased with gap distance (Fig. 6D). Both of these differences decrease to zero as the gap distance increases, suggesting that alignment between ground reaction force, touchdown velocity, and virtual leg angle increases as gap distance increases. In other words, the longer the gap distance, the more vector alignment is present, which could result in a reduction of joint moments.

Like squirrels, cats also align ground reaction forces on jump-down landings with their forelimbs (Zhang et al., 2014b). Specifically, cats land with shallower leg angles to offload GRF to their hindlimbs, thereby reducing the peak force on the forelimbs. The reduction protects the forelimbs from damage in higher speed landings. Wu et al. (2019) have shown that at higher jump heights, cat hindlimbs play a greater role than the forelimbs in absorbing landing energy as the body rotates down and the back bends to allow hindlimb touchdown. In fact, forelimb fractures (38.5%) are less common than hindlimb fractures (61.5%) in falling cats (Zhang et al., 2014b), highlighting the tendency to use the hindlimbs at higher drop heights. Therefore, in cats, posture-dependent actuation prior to touchdown allows the animal to tune the distribution of energy absorption between forelimbs and hindlimbs after touchdown.

EMG data show that cats have a generalized motor program that is independent to drop height and is used to activate extensor muscles at the elbow joint during the pre-landing phase of self-initiated jumps (McKinley and Smith, 1983; McKinley et al., 1983). In addition to pre-touchdown muscle activity and limb coordination, cats also possess passive, post-touchdown landing mechanisms. In particular, they show a remarkable multilevel energy buffering system for shock absorption that includes paw pads, limb bones, and coordinated joints complementing each other (Wu et al., 2020). These results have inspired the design of energy dissipation pads (Lu et al., 2021) and suggestions for legged landing robot design (Xu et al., 2022). Further definition of the complete energy buffering system used in squirrel landing will likely lead to additional inspiration.

In cane toads, Azizi et al. (2014) showed that rapid modulation of hindlimb flexion during the aerial phase of a hop could shift the COM anteriorly and reduce torque by aligning the COM with the GRF vector. A similar study using the same species of cane toads (*Bufo marinus*) performing controlled landings found that toads use their forelimbs exclusively to decelerate and stabilize the body after impact (Cox and Gillis, 2017). By observing animals jumping from platforms of different heights, they showed that toads achieve dynamic stability across a wide range of landing conditions. Specifically, Cox and Gillis (2017) found that torques during landing could be reduced by a landing preparation motor control strategy for aligning the forelimbs with the body's instantaneous velocity vector at impact (impact angle). As in our squirrels, these two toad studies together show the importance of CG alignment with both velocity and GRF vectors.

Energy absorption can also occur during flight prior to touchdown. In flying squirrels for example, landing force is negatively correlated with glide length (Byrnes et al., 2008). Longer glides allow more time for animals to reach body orientations where they can use aerodynamics to decrease landing velocity, and thus landing forces. In fact, Paskins et al. (2007) suggested flight in flying squirrels may have been selected to control landing forces. A study on birds by Provini et al. (2014) determined that the hindlimbs of zebra finches and diamond doves produce 1.4–2.6 BW forces during landing. It was estimated that for both species, the hindlimbs reduced landing velocity by 60%, thereby contributing substantially to the absorption of kinetic energy after touchdown. The flying robot SNAG (stereotyped nature-inspired aerial grasper) incorporated an independent passive energy absorption for each leg (Roderick et al., 2021). We surmise that the rich morphology of squirrel paws almost certainly contributes to passive energy management upon landing and deserves further attention.

### Landing stabilization by control of leg force and foot torque

Spring-loaded inverted pendulum (SLIP) models have long been used to test hypotheses for stable walking and running in animals (Blickhan and Full, 1993; Geyer et al., 2005; Seipel et al., 2004) and a whole host of robots (Raibert et al., 1986; McGeer, 1990). Squirrels, cats, anurans, and birds alike rely on their limbs to manage landing energy post-touchdown, regardless of their ability to control touchdown speed prior to touchdown, and therefore variations of spring-loaded inverted pendulum models could be useful to understand landing.

Zhang et al. (2014a) examined energy absorption and control by spring-mass modeling the front limb behavior of cats jumping down from 1.8 m high platforms onto a force plate. Toad (Cox and Gillis, 2017) and frog (Nauwelaerts and Aerts, 2006) landings after a hop used versions of spring-damper models to determine the alignment of forelimbs at impact and their angle for effective energy absorption. Birds have been studied using spring-mass models to design a flying robot that could perch on branches using an under-actuated, dynamic grasper (Roderick et al., 2021). Using a spring-mass model, the authors defined a ‘perching sufficiency region’. They found that the primary perching failure mode of slipping too far forward or back could be quantified by angular momentum about the branch, which is a function of mass distribution, velocity, and body angles relative to the perch (Roderick et al., 2021). To avoid toppling, balancers can generate force and torque.

Adding leg or radial force control to torque-based balance has been shown to expand disturbance rejection (Caron, 2020; Yang et al., 2021) and improve balance capture regions available to pendulum models (van Hofslot et al., 2019). Using balancing strategies with support forces and torques can also assist by adjusting linear or angular momentum (Orin et al., 2013; Lee and Goswami, 2012). We adopted the extensible pendulum model developed by Yim et al. (2025) for above-branch landing of the monopedal robot Salto. Yim et al. (submitted) proposed two adaptive control strategies that squirrels may be utilizing upon touchdown: leg force control and foot torque control.

Control hypotheses from Yim et al. (submitted) are summarized in Fig. 3B, and they are as follows. All else being equal, they postulate that when an overshoot landing error is present (Fig. 1, lightest red sector), an extensible pendulum can achieve balance by generating a greater leg force and a braking torque (Fig. 3B, overshoot). When an undershoot landing error occurs (Fig. 1, lightest blue sector), an extensible pendulum could achieve balance by producing a lesser leg force and a torque to pull up (Fig. 3B, undershoot). The extensible pendulum model was consistent with the robot's experimental data by predicting the effect of force and torque in the balanceable region. In the present study, we tested the control hypotheses proposed by Yim et al. (2025) by using the model to interpret our results on squirrels.

Testing the landing control hypothesis revealed that all else is not equal for the jumps at our three gap distances. Specifically, landing speed and velocity angle were significantly different at each gap distance, resulting in variation in both landing error and angular momentums (see Table 2). The larger variation in peak torque is likely because longer gap distances required higher take-off and landing speeds, which would yield greater variation observed in touchdown states, and consequently variation in leg force and foot torque. The space of stable above-branch landings is set by landing error and total angular momentum. A sequence of snapshots for a characteristic undershoot and overshoot trial are illustrated in Fig. 3A. The relationship between wrench and landing type indicates that fewer adjustments are necessary for a nominal landing (Fig. 1, white center sector) where the landing error is small and negative while the angular momentum is moderate. In this instance, a squirrel can passively use moderate post-impact angular momentum to compensate for the small, negative landing error, resulting in swinging up towards a balanced posture without the need for significant adjustments.

**Fig. 7.**
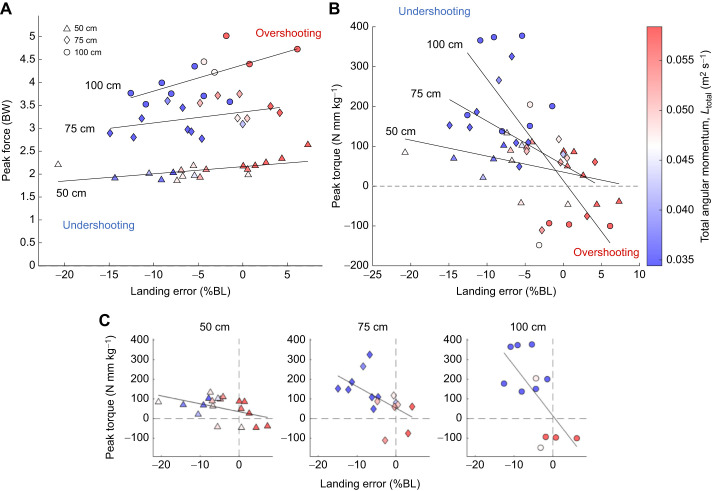
**Squirrel peak force data versus landing error and angular momentum as a function of gap distance.** (A) Squirrels tend to apply higher peak forces the more they overshoot (linear mixed-effects model controlling for gap and individual, *t*_1,45_=−3.4, *P*=0.0015). (B) Squirrel peak torque data versus landing error and angular momentum plotted for each gap distance. Squirrels tend to apply higher torque the more they undershoot (*t*_1,45_=−3.7, *P*<0.001). (C) Separate plots from B by gap distance (50 cm, *F*=4.8; 75 cm, *F*=6.5; 100 cm, *F*=8.6; all *P*<0.05).

However, when the magnitude of landing error increases, greater adjustments are necessary. For example, when the landing error is large and positive such that the ballistic trajectory of the CG is above the branch, total angular momentum is also high (Fig. 7A,B in red). Under these overshoot conditions, squirrels apply a large leg force (Fig. 7A) and a braking foot torque on the branch (Fig. 7B). According to the extensible pendulum model, a large leg force is useful for preventing leg shortening or even promoting leg lengthening, which would preserve or increase inertia. Increasing inertia about the branch would decrease angular velocity, resulting in a slower rotation that squirrels can use to avoid swinging over. Likewise, the landing trajectory of the CG could be significantly below the branch (large, negative landing error) and the total angular momentum could be small (Fig. 7A,B in blue). Given these undershoot conditions, squirrels tend to generate lower leg forces (Fig. 7A) and pull-up foot torques (Fig. 7B). According to the extensible pendulum model, a lesser leg force would result in faster leg shortening, which would decrease inertia. Decreasing inertia about the branch would increase angular velocity, resulting in a faster rotation that squirrels can use to swing up on the branch. We have no evidence to determine if squirrels activate muscles to generate additional torque with their feet alone, but grasping digits must provide necessary contact friction. Furthermore, at the gap distance of 100 cm, both peak force and torque seem to be most sensitive to landing error. In other words, for the same landing error but faster landing speed, even more adjustments in force and torque may be necessary for stable above-branch landing.

### Conclusion

In summary, our measurements on free-ranging squirrels shed light on their remarkable landing dynamics, uncovering findings that govern their agility and stability in arboreal environments. Along with general gap effects on landing kinetics, our investigation revealed three discoveries. First, squirrels exhibit rapid and precise landings, primarily utilizing their forelimbs to manage landing energy. Second, the alignment of velocity and force vectors along the limbs is more pronounced as landing speed increases, reflecting a possible adaptive strategy for managing landing energy. Third, the variability in peak force and torque is consistent with the control of overshooting and undershooting the landing target, suggesting squirrels use radial leg force and foot torque to adjust their landings actively. Specifically, squirrels employ substantial braking forces and torques when overshooting, while utilizing lesser leg force and pull-up torques to correct for undershooting.

Further exploration is warranted to deepen the understanding of squirrel landing dynamics. The extensible pendulum model and the cylindrical approximation of the squirrels' bodies used at touchdown do not include the variable curvature of the spine, the head, limbs and tail. A more complex model might reveal additional mechanisms for balancing moments and be useful for testing hypotheses about the role of the variable body and appendage inertia. XROMM data could better reveal the utility of modeling additional degrees of freedom. One example could be in revealing the stabilizing function of the shoulder and back, as suggested in cats (Zhang et al., 2014a). Kinematic analyses encompassing the entire time series could elucidate the changes in leg length over time, offering valuable insights into how leg force adjustments contribute to leg length changes and the correction of landing error. Additionally, investigating the effects of foothold parameters such as size, curvature and friction on landing control could provide a comprehensive perspective on the adaptability of these findings in different environments.

The implications of the present findings extend beyond the realm of squirrel biomechanics. Advancements in the field of aerial robotics have demonstrated robot abilities such as landing dynamically and perching on cylindrical substrates using specialized grippers (Roderick et al., 2021; Zufferey et al., 2022; Chen et al., 2022; Thomas et al., 2013). These specialized mechanisms ensure anchoring to the substrate and enhance stability during critical landing phases. Specialized gripper designs could be integrated into quadruped robots, which have demonstrated dynamic capabilities such as walking across bricks (Agrawal et al., 2022), walking along a thin walkway (Gonzalez et al., 2020) and even jumping/landing optimally on flat surfaces (Nguyen et al., 2019; Jeon et al., 2022). However, implementation of foot designs more complex than wheels or spheres remains an open challenge owing to the complexity of modeling multiple contacts for legged robot control.

One way we can complement and simplify control is by designing passive feet using compliant structures that react to substrate forces such as bistable mechanisms (Jin et al., 2023), finray designs (Manoonpong et al., 2022; Rozen-Levy et al., 2021) and multi-segment tendon-driven feet (Chatterjee et al., 2023). Our latest work examines the effect that stiffness and damping have on tendon-driven feet for dynamic branch landing (Wang et al., 2024). These passive foot designs simplify legged robot control, and they have the potential for becoming useful for dynamic grasping and detachment for agile robot locomotion on sparse terrains. Exploring the scalability of these control strategies from diverse biological systems holds promise, particularly in the domain of quadruped robot locomotion. The landing mechanisms inherent to squirrels, manifested in their body, limbs and feet, could provide inspiration for the design and control of innovative, agile, legged robots equipped with the ability to rapidly traverse sparse terrains for societal benefit.

## Supplementary Material

10.1242/jeb.249934_sup1 Supplementary information

Dataset 1. Extracted features from kinematics and force-torque data for each trial. This dataset contains extracted features described in the Feature Extraction section of Methods tabulated for each trial. The dataset is structure by trial, with each row representing one trial and each column representing a specific extracted feature.

## References

[JEB249934C1] Agrawal, A., Chen, S., Rai, A. and Sreenath, K. (2022). Vision-aided dynamic quadrupedal locomotion on discrete terrain using motion libraries. In: 2022 International Conference on Robotics and Automation (ICRA), May (pp. 4708-4714). IEEE.

[JEB249934C2] Alexander, R. M. (1991). Energy-saving mechanisms in walking and running. *J. Exp. Biol.* 160, 55-69. 10.1242/jeb.160.1.55 1960518

[JEB249934C3] Azizi, E., Larson, N. P., Abbott, E. M. and Danos, N. (2014). Reduce torques and stick the landing: limb posture during landing in toads. *J. Exp. Biol.* 217, 3742-3747. 10.1242/jeb.108506 25320271

[JEB249934C4] Blickhan, R. and Full, R. J. (1993). Similarity in multilegged locomotion: bouncing like a monopode. *J. Comp. Physiol. A* 173, 509-517. 10.1007/BF00197760

[JEB249934C5] Bonser, R. H. (1999). Branching out in locomotion: the mechanics of perch use in birds and primates. *J. Exp. Biol.* 202, 1459-1463. 10.1242/jeb.202.11.1459 10229692

[JEB249934C6] Boulinguez-Ambroise, G., Dunham, N., Phelps, T., Mazonas, T., Nguyen, P., Bradley- Cronkwright, M., Boyer, D. M., Yapuncich, G. S., Zeininger, A., Schmitt, D. et al. (2023). Jumping performance in tree squirrels: insights into primate evolution. *J. Hum. Evol.* 180, 103386. 10.1016/j.jhevol.2023.103386 37209637

[JEB249934C7] Byrnes, G., Lim, N. T. L. and Spence, A. J. (2008). Take-off and landing kinetics of a free-ranging gliding mammal, the Malayan colugo (*Galeopterus variegatus*). *Proc. R. Soc. B* 275, 1007-1013. 10.1098/rspb.2007.1684 PMC2600906 18252673

[JEB249934C8] Caron, S. (2020). Biped stabilization by linear feedback of the variable-height inverted pendulum model. In: 2020 IEEE International Conference on Robotics and Automation (ICRA), May (pp. 9782-9788). IEEE.

[JEB249934C9] Chatterjee, A., Mo, A., Kiss, B., Gönen, E. C. and Badri-Spröwitz, A. (2023). Multi-segmented adaptive feet for versatile legged locomotion in natural terrain. In: 2023 IEEE International Conference on Robotics and Automation (ICRA), May (pp. 1162-1169). IEEE.

[JEB249934C10] Chen, J. J., Peattie, A. M., Autumn, K. and Full, R. J. (2006). Differential leg function in a sprawled-posture quadrupedal trotter. *J. Exp. Biol.* 209, 249-259. 10.1242/jeb.01979 16391347

[JEB249934C11] Chen, T. G., Hoffmann, K. A., Low, J. E., Nagami, K., Lentink, D. and Cutkosky, M. R. (2022). Aerial grasping and the velocity sufficiency region. *IEEE Robot. Autom. Lett.* 7, 10009-10016. 10.1109/LRA.2022.3192652

[JEB249934C12] Cox, S. M. and Gillis, G. (2017). Evidence toads may modulate landing preparation without predicting impact time. *Biol. Open* 6, 71-76. 10.1242/bio.022707 27895052 PMC5278434

[JEB249934C13] Cutkosky, M. R. and Howe, R. D. (1990). Human grasp choice and robotic grasp analysis. In *Dextrous Robot Hands* (ed. S. T. Venkatataman and T. Iberall), pp. 5-31. Springer.

[JEB249934C14] Demes, B., Jungers, W. L., Gross, T. S. and Fleagle, J. G. (1995). Kinetics of leaping primates: influence of substrate orientation and compliance. *Am. J. Phys. Anthropol.* 96, 419-429. 10.1002/ajpa.1330960407 7604894

[JEB249934C15] Demes, B., Fleagle, J. G. and Jungers, W. L. (1999). Takeoff and landing forces of leaping strepsirrhine primates. *J. Hum. Evol.* 37, 279-292. 10.1006/jhev.1999.0311 10444353

[JEB249934C16] Demes, B., Franz, T. M. and Carlson, K. J. (2005). External forces on the limbs of jumping lemurs at takeoff and landing. *Am. J. Phys. Anthropol.* 128, 348-358. 10.1002/ajpa.20043 15810009

[JEB249934C17] Dunham, N. T., McNamara, A., Shapiro, L., Phelps, T., Wolfe, A. N. and Young, J. W. (2019). Locomotor kinematics of tree squirrels (Sciurus carolinensis) in free-ranging and laboratory environments: implications for primate locomotion and evolution. *J. Exp. Zool. A Ecol. Integr. Physiol.* 331, 103-119. 10.1002/jez.2242 30369092

[JEB249934C18] Essner, R. L. Jr. (2002). Three-dimensional launch kinematics in leaping, parachuting and gliding squirrels. *J. Exp. Biol.* 205, 2469-2477. 10.1242/jeb.205.16.2469 12124370

[JEB249934C19] Feix, T., Romero, J., Schmiedmayer, H. B., Dollar, A. M. and Kragic, D. (2015). The grasp taxonomy of human grasp types. *IEEE Trans. Hum. Mach. Syst.* 46, 66-77. 10.1109/THMS.2015.2470657

[JEB249934C20] Full, R. J., Blickhan, R. and Ting, L. H. (1991). Leg design in hexapedal runners. *J. Exp. Biol.* 158, 369-390. 10.1242/jeb.158.1.369 1919412

[JEB249934C21] Geyer, H., Seyfarth, A. and Blickhan, R. (2005). Spring-mass running: simple approximate solution and application to gait stability. *J. Theor. Biol.* 232, 315-328. 10.1016/j.jtbi.2004.08.015 15572057

[JEB249934C22] Gonzalez, C., Barasuol, V., Frigerio, M., Featherstone, R., Caldwell, D. G. and Semini, C. (2020). Line walking and balancing for legged robots with point feet. In: 2020 IEEE/RSJ International Conference on Intelligent Robots and Systems (IROS), October (pp. 3649-3656). IEEE.

[JEB249934C23] Graham, M. and Socha, J. J. (2020). Going the distance: the biomechanics of gap-crossing behaviors. *J. Exp. Zool. Part A Ecol. Integr. Physiol.* 333, 60-73. 10.1002/jez.2266 31111626

[JEB249934C24] Haldane, D. W., Plecnik, M. M., Yim, J. K. and Fearing, R. S. (2016). Robotic vertical jumping agility via series-elastic power modulation. *Sci. Robot.* 1, eaag2048. 10.1126/scirobotics.aag2048 33157854

[JEB249934C25] Hesse, B., Nyakatura, J. A., Fischer, M. S. and Schmidt, M. (2015). Adjustments of limb mechanics in cotton-top tamarins to moderate and steep support orientations: significance for the understanding of early primate evolution. *J. Mamm. Evol.* 22, 435-450. 10.1007/s10914-014-9283-4

[JEB249934C26] Hunt, N. H., Jinn, J., Jacobs, L. F. and Full, R. J. (2021). Acrobatic squirrels learn to leap and land on tree branches without falling. *Science* 373, 697-700. 10.1126/science.abe5753 34353955 PMC9446516

[JEB249934C27] Jeon, S. H., Kim, S. and Kim, D. (2022). Online optimal landing control of the MIT mini cheetah. In: 2022 International Conference on Robotics and Automation (ICRA), May (pp. 178-184). IEEE.

[JEB249934C28] Jin, L., Yang, Y., Maldonado, B. O., Lee, S. D., Figueroa, N., Full, R. J. and Yang, S. (2023). Ultra-fast, programmable, and electronics-free soft robots enabled by snapping metacaps. *Adv. Intell. Syst.* 5, 2300039. 10.1002/aisy.202300039

[JEB249934C29] Lammers, A. R. and Gauntner, T. (2008). Mechanics of torque generation during quadrupedal arboreal locomotion. *J. Biomech.* 41, 2388-2395. 10.1016/j.jbiomech.2008.05.038 18619599

[JEB249934C30] Lee, S. H. and Goswami, A. (2012). A momentum-based balance controller for humanoid robots on non-level and non-stationary ground. *Auton. Robots* 33, 399-414. 10.1007/s10514-012-9294-z

[JEB249934C31] Lu, W., Zhang, Q., Qin, F., Xu, P., Chen, Q., Wang, H., Scarpa, F. and Peng, H. X. (2021). Hierarchical network structural composites for extraordinary energy dissipation inspired by the cat paw. *Appl. Mater. Today.* 25, 101222. 10.1016/j.apmt.2021.101222

[JEB249934C32] Manoonpong, P., Rajabi, H., Larsen, J. C., Raoufi, S. S., Asawalertsak, N., Homchanthanakul, J., Tramsen, H. T., Darvizeh, A. and Gorb, S. N. (2022). Fin ray crossbeam angles for efficient foot design for energy–efficient robot locomotion. *Adv. Intell. Syst.* 4, 2100133. 10.1002/aisy.202100133

[JEB249934C33] McGeer, T. (1990). Passive dynamic walking. *Int. J. Robot. Res.* 9, 62-82. 10.1177/027836499000900206

[JEB249934C34] McKinley, P. A. and Smith, J.,L. (1983). Visual and vestibular contributions to prelanding EMG during jump-downs in cats. *Exp. Brain Res.* 52, 439-448. 10.1007/BF00238037 6606584

[JEB249934C35] McKinley, P. A., Smith, J. L. and Gregor, R. J. (1983). Responses of elbow extensors to landing forces during jump downs in cats. *Exp. Brain Res.* 49, 218-228. 10.1007/BF00238582 6832259

[JEB249934C36] Nauwelaerts, S. and Aerts, P. (2006). Take-off and landing forces in jumping frogs. *J. Exp. Biol.* 209, 66-77. 10.1242/jeb.01969 16354779

[JEB249934C37] Nguyen, Q., Powell, M. J., Katz, B., Di Carlo, J. and Kim, S. (2019). Optimized jumping on the mit cheetah 3 robot. In: 2019 International Conference on Robotics and Automation (ICRA), May (pp. 7448-7454). IEEE.

[JEB249934C38] Nyakatura, J. A. (2019). Early primate evolution: insights into the functional significance of grasping from motion analyses of extant mammals. *Biol. J. Linn. Soc.* 127, 611-631. 10.1093/biolinnean/blz057

[JEB249934C39] Orin, D. E., Goswami, A. and Lee, S.-H. (2013). Centroidal dynamics of a humanoid robot. *Auton. Robots* 35, 161-176. 10.1007/s10514-013-9341-4

[JEB249934C40] Orkin, J. D. and Pontzer, H. (2011). The narrow niche hypothesis: gray squirrels shed new light on primate origins. *Am. J. Phys. Anthropol.* 144.4, 617-624. 10.1002/ajpa.21450 21404237

[JEB249934C41] Paskins, K. E., Bowyer, A., Megill, W. M. and Scheibe, J. S. (2007). Take-off and landing forces and the evolution of controlled gliding in northern flying squirrels glaucomys sabrinus. *J. Exp. Biol.* 210, 1413-1423. 10.1242/jeb.02747 17401124

[JEB249934C42] Pouydebat, E., Boulinguez-Ambroise, G., Manzano, A., Abdala, V. and Sustaita, D. (2023). Convergent evolution of manual and pedal grasping capabilities in tetrapods. In *Convergent Evolution: Animal Form and Function* (ed. V. L. Bels and A. P. Russell), pp. 323-389. Cham: Springer International Publishing.

[JEB249934C43] Provini, P., Tobalske, B. W., Crandell, K. E. and Abourachid, A. (2014). Transition from wing to leg forces during landing in birds. *J. Exp. Biol.* 217, 2659-2666. 10.1242/jeb.104588 24855670

[JEB249934C44] Raibert, M. H., Chepponis, M. and Brown, J. H. B. (1986). Running on four legs as though they were one. *IEEE J. Robot. Autom.* 2, 70-82. 10.1109/JRA.1986.1087044

[JEB249934C45] Reilly, S. M., Montuelle, S. J., Schmidt, A., Naylor, E., Jorgensen, M. E., Halsey, L. G. and Essner, R. L. Jr. (2015). Conquering the world in leaps and bounds: hopping locomotion in toads is actually bounding. *Funct. Ecol.* 29, 1308-1316. 10.1111/1365-2435.12414

[JEB249934C46] Roderick, W. R., Chin, D. D., Cutkosky, M. R. and Lentink, D. (2019). Birds land reliably on complex surfaces by adapting their foot-surface interactions upon contact. *eLife* 8, e46415. 10.7554/eLife.46415 31385573 PMC6684272

[JEB249934C47] Roderick, W. R., Cutkosky, M. R. and Lentink, D. (2021). Bird-inspired dynamic grasping and perching in arboreal environments. *Sci. Robot.* 6, eabj7562. 10.1126/scirobotics.abj7562 34851710

[JEB249934C48] Rozen-Levy, S., Messner, W. and Trimmer, B. A. (2021). The design and development of branch bot: a branch-crawling, caterpillar-inspired, soft robot. *Int. J. Robot. Res.* 40, 24-36. 10.1177/0278364919846358

[JEB249934C49] Scheibe, J. S., Paskins, K. E., Ferdous, S. and Birdsill, D. (2007). Kinematics and functional morphology of leaping, landing, and branch use in *Glaucomys sabrinus*. *J. Mammal.* 88, 850-861. 10.1644/06-MAMM-S-331R1.1

[JEB249934C50] Schmidt, A. (2011). Functional differentiation of trailing and leading forelimbs during locomotion on the ground and on a horizontal branch in the European red squirrel (*Sciurus vulgaris*, Rodentia). *Zoology* 114.3, 155-164. 10.1016/j.zool.2011.01.001 21658923

[JEB249934C51] Schmidt, A. and Fischer, M. S. (2011). The kinematic consequences of locomotion on sloped arboreal substrates in a generalized (*Rattus norvegicus*) and a specialized (*Sciurus vulgaris*) rodent. *J. Exp. Biol.* 214, 2544-2559. 10.1242/jeb.051086 21753049

[JEB249934C52] Seipel, J. E., Holmes, P. J. and Full, R. J. (2004). Dynamics and stability of insect locomotion: a hexapedal model for horizontal plane motions. *Biol. Cybern.* 91, 76-90. 10.1007/s00422-004-0498-y 15322851

[JEB249934C53] Sustaita, D., Pouydebat, E., Manzano, A., Abdala, V., Hertel, F. and Herrel, A. (2013). Getting a grip on tetrapod grasping: form, function, and evolution. *Biol. Rev.* 88, 380-405. 10.1111/brv.12010 23286759

[JEB249934C54] Thomas, J., Polin, J., Sreenath, K. and Kumar, V. (2013). Avian-inspired grasping for quadrotor micro UAVs. In: International Design Engineering Technical Conferences and Computers and Information in Engineering Conference, August (Vol. 55935, p. V06AT07A014). American Society of Mechanical Engineers.

[JEB249934C55] Toussaint, S., Llamosi, A., Morino, L. and Youlatos, D. (2020). The central role of small vertical substrates for the origin of grasping in early primates. *Curr. Biol.* 30, 1600-1613. 10.1016/j.cub.2020.02.012 32169214

[JEB249934C56] van Hofslot, B. J., Griffin, R., Bertrand, S. and Pratt, J. (2019). Balancing using vertical center-of-mass motion: a 2-D analysis from model to robot. *IEEE Robot. Autom. Lett.* 4, 3247-3254. 10.1109/LRA.2019.2925303

[JEB249934C57] Wang, S., Kuang, D., Lee, S. D., Full, R. J. and Stuart, H. S. (2024). Squirrel-inspired tendon-driven passive gripper for agile landing. In: IEEE International Conference on Robotics and Automation (ICRA), May 13 (pp. 4184-4190).

[JEB249934C58] Wölfer, J., Aschenbach, T., Michel, J. and Nyakatura, J. A. (2021). Mechanics of arboreal locomotion in swinhoe's striped squirrels: a potential model for early euarchontoglires. *Front. Ecol. Evol.* 9, 636039. 10.3389/fevo.2021.636039

[JEB249934C59] Wu, X., Pei, B., Pei, Y., Wu, N., Zhou, K., Hao, Y. and Wang, W. (2019). Contributions of limb joints to energy absorption during landing in cats. *Appl. Bionics Biomech.* 2019, 3815612. 10.1155/2019/3815612 31531125 PMC6721424

[JEB249934C60] Wu, X., Pei, B., Pei, Y., Wang, W., Hao, Y. and Zhou, K. (2020). How do cats resist landing injury: insights into the multi-level buffering mechanism. *J. Bionic Eng.* 17, 600-610. 10.1007/s42235-020-0048-x

[JEB249934C61] Xu, D., Zhou, H., Jiang, X., Li, S., Zhang, Q., Baker, J. S. and Gu, Y. (2022). New insights for the design of bionic robots: adaptive motion adjustment strategies during feline landings. *Front. Vet. Sci.* 9, 836043. 10.3389/fvets.2022.836043 35529841 PMC9070819

[JEB249934C62] Yang, S., Chen, H., Zhang, L., Cao, Z., Wensing, P. M., Liu, Y., Pang, J. and Zhang, W. (2021). Reachability-based push recovery for humanoid robots with variable-height inverted pendulum. In: 2021 IEEE International Conference on Robotics and Automation (ICRA), May 30 (pp. 3054-3060). IEEE.

[JEB249934C63] Yim, J. K. and Fearing, R. S. (2018). Precision jumping limits from flight-phase control in salto-1p. In: 2018 IEEE/RSJ international conference on intelligent robots and systems (IROS), pp. 2229-2236. IEEE.

[JEB249934C64] Yim, J. K., Singh, B. R. P., Wang, E. K., Featherstone, R. and Fearing, R. S. (2020). Precision robotic leaping and landing using stance-phase balance. *IEEE Robot. Autom. Lett.* 5, 3422-3429. 10.1109/LRA.2020.2976597

[JEB249934C65] Yim, J. K., Wang, E. K., Lee, S. D., Hunt, N. H., Full, R. J. and Fearing, R. S. (2025). Monopedal robot branch-to-branch leaping and landing inspired by squirrel balance control. *Sci. Robot.* 10, eadq1949. 10.1126/scirobotics.adq1949 40106660

[JEB249934C66] Youlatos, D. (1999). Locomotor and postural behavior of *Sciurus igniventris* and Microsciurus flaviventer (Rodentia, Sciuridae) in eastern Ecuador. *Mammalia* 63, 405-416. 10.1515/mamm.1999.63.4.405

[JEB249934C67] Young, J. W. (2023). Convergence of arboreal locomotor specialization: morphological and behavioral solutions for movement on narrow and compliant supports. In *Convergent Evolution: Animal Form and Function* (ed. V. L. Bels and A. P. Russell), pp. 289-322. Cham: Springer International Publishing.

[JEB249934C68] Young, J. W. and Chadwell, B. A. (2020). Not all fine-branch locomotion is equal: grasping morphology determines locomotor performance on narrow supports. *J. Hum. Evol.* 142, 1027. 10.1016/j.jhevol.2020.102767 32240883

[JEB249934C69] Zhang, Z., Yang, J. and Yu, H. (2014a). Effect of flexible back on energy absorption during landing in cats: a biomechanical investigation. *J. Bionic Eng.* 11, 506-516. 10.1016/S1672-6529(14)60063-9

[JEB249934C70] Zhang, Z., Yu, H., Yang, J., Wang, L. and Yang, L. (2014b). How cat lands: insights into contribution of the forelimbs and hindlimbs to attenuating impact force. *Chin. Sci. Bull.* 59, 3325-3332. 10.1007/s11434-014-0328-0

[JEB249934C71] Zufferey, R., Tormo-Barbero, J., Feliu-Talegón, D., Nekoo, S. R., Acosta, J. Á. and Ollero, A. (2022). How ornithopters can perch autonomously on a branch. *Nat. Commun.* 13, 771. 10.1038/s41467-022-35356-5 36513661 PMC9747916

