# Supplementary material for: Free-ranging squirrels perform stable, above-branch landings by balancing using leg force and nonprehensile foot torque
Source: J Exp Biol. 2025 Apr 4;228(7):jeb249934. doi: 10.1242/jeb.249934 (PMC11993264; doi:10.1242/jeb.249934)
Supplement: Supplementary information [file jexbio-228-249934-s1.pdf]

Dataset 1. Extracted features from kinematics and force-torque data for each trial. This dataset contains extracted features described in the Feature Extraction section of Methods tabulated for each trial. The dataset is structure by trial, with each row representing one trial and each column representing a specific extracted feature.

Available for download at
https://journals.biologists.com/jeb/article-lookup/doi/10.1242/jeb.249934#supplementary-data

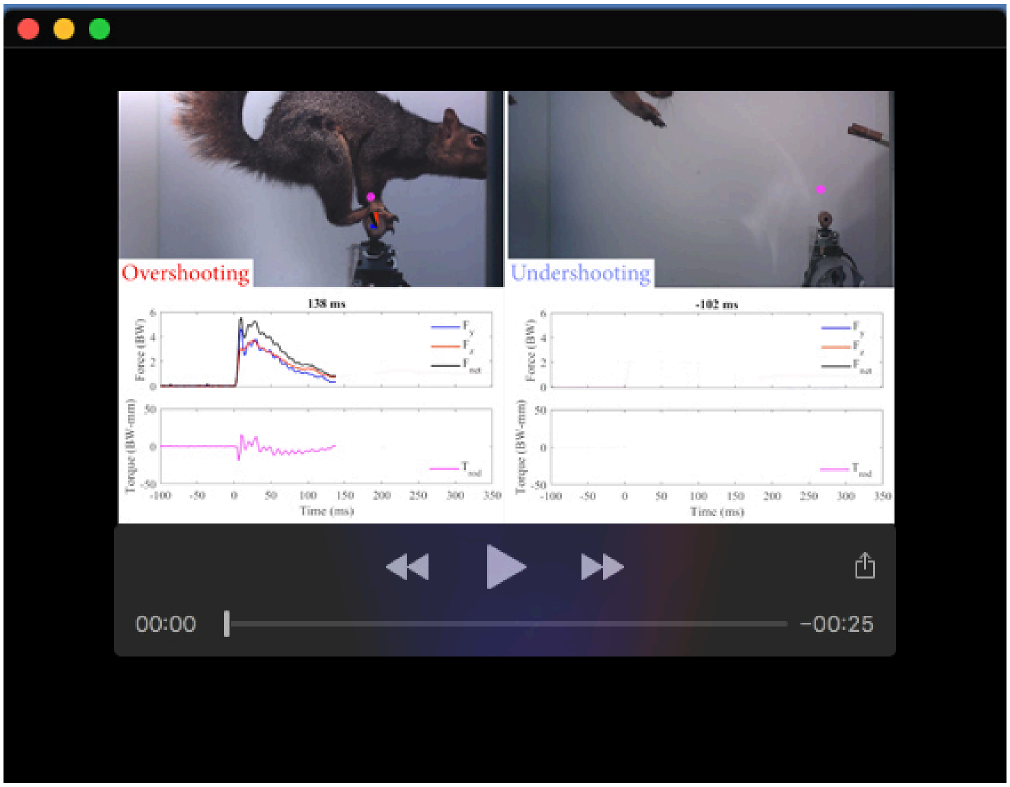

Movie 1. Side-by-side videos of squirrel undershooting and overshooting with corresponding branch reaction force and torque. Force and torque are plotted and synced with corresponding videos for an undershooting case and an overshooting case. When undershooting, the squirrel applies high pull-up torque throughout the landing. When overshooting on the other hand, the squirrel applies low and even negative braking torque while simultaneously applying higher leg braking force.

Journal of Experimental Biology • Supplementary information
